# Causality between inflammatory bowel disease and the cerebral cortex: insights from Mendelian randomization and integrated bioinformatics analysis

**DOI:** 10.3389/fimmu.2023.1175873

**Published:** 2023-07-27

**Authors:** Shubei He, Ying Peng, Xiaofang Chen, Ying Ou

**Affiliations:** ^1^ Department of Gastroenterology, The First Affiliated Hospital (Southwest Hospital) to Third Military Medical University (Army Medical University), Chongqing, China; ^2^ Institute of Digestive Diseases of the People's Liberation Army, The First Affiliated Hospital (Southwest Hospital) to Third Military Medical University (Army Medical University), Chongqing, China; ^3^ Cholestatic Liver Diseases Center, The First Affiliated Hospital (Southwest Hospital) to Third Military Medical University (Army Medical University), Chongqing, China; ^4^ Center for Metabolic Associated Fatty Liver Disease, The First Affiliated Hospital (Southwest Hospital) to Third Military Medical University (Army Medical University), Chongqing, China; ^5^ Department of Psychiatry, The Affiliated Hospital of Southwest Medical University, Luzhou, China

**Keywords:** inflammatory bowel disease, cerebral cortex, causality, Mendelian randomization, integrated bioinformatics

## Abstract

**Background:**

Inflammatory bowel disease (IBD), which includes ulcerative colitis (UC) and Crohn’s disease (CD), is a chronic, progressive, and recurrent intestinal condition that poses a significant global health burden. The high prevalence of neuropsychiatric comorbidities in IBD necessitates the development of targeted management strategies.

**Methods:**

Leveraging genetic data from genome-wide association studies and Immunochip genotype analyses of nearly 150,000 individuals, we conducted a two-sample Mendelian randomization study to elucidate the driving force of IBD, UC, and CD on cortical reshaping. Genetic variants mediating the causality were collected to disclose the biological pathways linking intestinal inflammation to brain dysfunction.

**Results:**

Here, 115, 69, and 98 instrumental variables genetically predicted IBD, UC, and CD. We found that CD significantly decreased the surface area of the temporal pole gyrus (β = −0.946 mm^2^, *P* = 0.005, false discovery rate-*P* = 0.085). Additionally, we identified suggestive variations in cortical surface area and thickness induced by exposure across eight functional gyri. The top 10 variant-matched genes were *STAT3*, *FOS*, *NFKB1*, *JAK2*, *STAT4*, *TYK2*, *SMAD3*, *IL12B*, *MYC*, *and CCL2*, which are interconnected in the interaction network and play a role in inflammatory and immune processes.

**Conclusion:**

We explore the causality between intestinal inflammation and altered cortical morphology. It is likely that neuroinflammation-induced damage, impaired neurological function, and persistent nociceptive input lead to morphological changes in the cerebral cortex, which may trigger neuropsychiatric disorders.

## 1 Introduction

Inflammatory bowel disease (IBD) encompasses a range of chronic nonspecific inflammatory disorders affecting the intestine and extraintestinal organs (1). The two primary phenotypes of IBD are ulcerative colitis (UC) and Crohn’s disease (CD), which are differentiated based on clinical characteristics. Historically, IBD has been predominantly observed in individuals of European descent. However, there has been a significant increase in the incidence of IBD among Americans and non-white populations, leading to a global health burden (2).

More than 20% of IBD patients suffer from mental disorders (3), while the prevalence of combined neurodegenerative diseases is at least 1.14-fold higher than that in healthy populations or patients with other long-standing illnesses (4). The correlation between chronic gut inflammation and neuropsychologic abnormalities has been proactively initiated these years, spawning the concept of the gut–brain axis—a bidirectional communication network between the intestine and the brain (5, 6). Animal experiments have demonstrated reduced neurogenesis and inhibited neuronal progenitor proliferation in mice with induced colitis (7, 8). Similarly, clinical trials targeting IBD populations with neuropsychiatric symptoms revealed regionally morphologic changes in the gray matter, making them promising neuroimaging biomarkers (9, 10). Taken together, these findings suggest that IBD can trigger structural variations in the brain, particularly in the cerebral cortex, which is the foundation for advanced neural activity. However, there are still gaps in our understanding of the causal relationship between IBD and the cerebral cortex due to incomplete translation of animal models to humans. Moreover, limited sample sizes and confounding factors in clinical cohorts present challenges when drawing definitive conclusions.

As an epidemiologic approach, Mendelian randomization (MR) allows for assessing causal relationships between specific exposure and outcome traits by randomly allocating genetic variants as instrumental variables (11, 12), which is applicable in etiological studies. Recently, MR has been employed to identify genetic overlap between intestinal inflammation and brain function (13). The application of MR in the field of the gut–brain axis has yielded valuable insights into potential therapeutic targets for related diseases and has sparked further investigations. Compared with traditional observations and randomized controlled trials, MR eliminates the drawbacks of potential confounding effects, inverse causality, and high execution difficulty.

Using publicly available genome-wide association studies (GWASs), we conducted a two-sample MR analysis to investigate the effect of IBD, UC, and CD on the cortical structure. This study aims to identify underlying factors that contribute to neuropsychiatric symptoms in patients with IBD and facilitate the discovery of the gut–brain axis. By exploring the mechanisms underlying cortical structural modifications, we hope to gain new insights that can inform the development of pharmaceutical therapies.

## 2 Methods

### 2.1 Data sources

#### 2.1.1 Exposure: inflammatory bowel disease

The GWAS summary statistics for IBD, UC, and CD were obtained from a trans-ethnic association study conducted by Liu et al. (14), who aggregated GWASs and Immunochip genotype data from a total of 86,640 European participants (38,155 cases; 48,485 controls) as well as 9,846 non-European ancestry participants (**Supplementary Table S1**). We downloaded the compiled information from the NHGRI-EBI GWAS catalog (15), which serves as a comprehensive repository of freely accessible bioinformatics resources.

#### 2.1.2 Outcome: cerebral cortex structure

The GWAS summary statistics of the cerebral cortex were derived from a structural magnetic resonance imaging (MRI)-based study that included 51,665 European individuals (**Supplementary Table S2**). Grasby et al. (16) comprehensively analyzed the entire cortex. They developed 138 distinctive phenotypes [total surface area (SA) and average thickness (TH), 34 globally controlled cortical SA and TH, 34 non-globally controlled cortical SA and TH]. We employed the GWAS data for these 138 cortical traits to explore whether IBD, UC, and CD causally induce cortical structural changes.

Our study only extracted GWAS summary statistics from published studies, which were also publicly accessible. No extra ethical approval or informed consent was required.

### 2.2 Selection of genetic instruments

**Figure 1:**
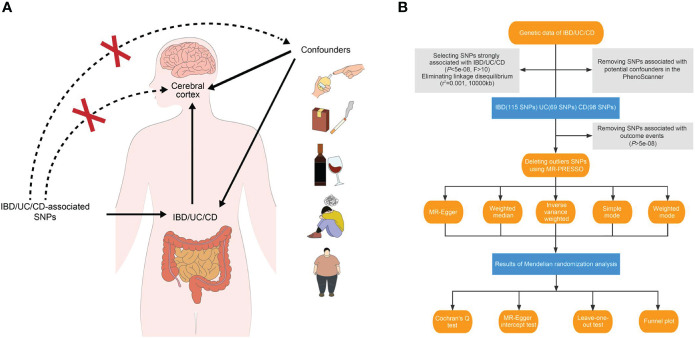
Study design overview. **(A)** Three essential hypotheses of Mendelian randomization in our study. **(B)** Flowchart of Mendelian randomization analysis disclosing the underlying role of IBD, UC, and CD in the altered brain structure. IBD, inflammatory bowel disease; UC, ulcerative colitis; CD, Crohn’s disease; SNP, single nucleotide polymorphism.

In essence, MR involves using a genome-wide single nucleotide polymorphism (SNP) as an instrumental variable to validate the exposure–outcome association. The beta values and standard errors of SNPs for each trait are selected for MR analysis. A qualified instrumental variant must adhere to the following principles (11): 1) it should be strongly correlated with the exposure data at a test threshold of 5 × 10^−8^; 2) it should not be directly associated with the outcome trait, indicating that any observed causality is solely driven by the exposure; and 3) it should independently establish exposure–outcome causality, excluding the influence of confounding factors (**Figure 1A**).

For our study, we applied three main hypotheses to select genetic variants (**Figure 1B**). Firstly, we excluded SNPs that did not reach genome-wide significance at a threshold of 5 × 10^−8^ in the respective summarized dataset for the exposure phenotypes. We also removed SNPs in high linkage disequilibrium with an r^2^ value of 0.001 and a physical distance of 10,000 kb from the index variant. Additionally, we calculated F-statistics for candidate SNPs and retained those with an F-statistic exceeding 10 (17, 18). After incorporating the outcome data, instruments significantly associated with the cerebral cortex at a significance level of 5 × 10^−8^ were excluded.

To establish a direct causal IBD–brain relationship, we accounted for potential confounding factors such as obesity, body mass index, type 1 and type 2 diabetes mellitus, hypertension, smoking, drinking, and neuropsychiatric diseases. We employed PhenoScanner, an online database of human genotype–phenotype associations (www.phenoscanner.medschl.cam.ac.uk), to identify and exclude confounder SNPs from the genetic variants (*P*-value: 1 × 10^-5^, r^2^: 0.8, European, reference: GRCh37).

### 2.3 Analyses of causal effect

Outlier SNPs should be identified and removed using the MR-PRESSO method prior to conducting the MR analysis. In our study, a two-sample MR analysis was employed to extrapolate the extent and direction of the effect of IBD on brain structure. Five strategies were utilized: MR-Egger, random-effect inverse-variance weighted (IVW), weighted median, simple mode, and weighted mode. The MR-Egger method accommodates the presence of horizontal pleiotropy from genetic variants, provided that it is uncorrelated with the instrument–exposure correlation (19). The weighted median-based MR approach remains unbiased as long as at least half of the instrumental variables are non-pleiotropic (20). The mode-based approaches cluster similar variants and derive estimates based on the specific aggregation with the highest number of SNPs (21). We have opted to endorse the IVW method among these approaches, as it conducts a meta-analysis of the Wald ratio across all instrumental SNPs (22). Meanwhile, if the results from the remaining four methods are consistent with those generated by IVW, they can be employed to supplement IVW estimates.

Once causality is established, heterogeneity and pleiotropy tests are required to confirm the findings’ reliability. The estimate is non-heterogenetic if the *P*-value from Cochran’s Q test exceeds 0.05. MR-Egger intercept test (*P*-value threshold of 0.05) and leave-one-out plot are utilized to verify the presence of horizontal pleiotropy. Additionally, a funnel plot evaluates if directional pleiotropy is presented. To reinforce the credibility of our results, we employed an online calculator to assess the power of MR analyses (https://sb452.shinyapps.io/power/).

### 2.4 Function exploration of mediator genes

We curated and consolidated mediator instruments that establish a connection between IBD and the cerebral cortex. Open Targets Genetics (https://genetics.opentargets.org/) is a variant-centric tool that integrates functional genomics data and quantitative trait loci from multiple heterogeneous sources to generate overall variant-to-gene (V2G) scores, which enables us to prioritize candidate genes. Genes with the highest overall V2G scores were selected and annotated using Gene Ontology (GO) and Kyoto Encyclopedia of Genes and Genomes (KEGG) pathway enrichment analyses. Those pathways with less than five genes and more than 500 genes should be removed before the enrichment analysis.

Using bioinformatics approaches, we further established interaction networks and tracked hub nodes that regulate other genes in various physiological processes. These efforts were valuable in detecting shared loci with the cerebral cortex in several loci confirmed to be associated with IBD, thereby providing fresh insights into targeted therapies.

### 2.5 Statistical analysis

R studio (version 4.1.2) was utilized for conducting MR analyses and function annotation. The TwoSampleMR package (version 0.5.6) was employed for the MR analysis, while org.Hs.eg.db (version 3.16.0) and clusterProfiler (version 4.2.2) packages were used for species annotation and subsequent pathway enrichment.

To address the issue of repeated calculations within the same datasets, we implemented the Benjamini–Hochberg procedure to control the false discovery rate (FDR). Significant estimates were those with a *P*-value < 0.05 and an FDR-adjusted *P*-value < 0.1. We considered results with a *P*-value < 0.05 but an FDR-adjusted *P*-value > 0.1 to be suggestive in nature.

Protein–protein interactions were investigated using STRING (Search Tool for the Retrieval of Interacting Genes), a database of known and predicted interactions between proteins. The complex networks were visualized and integrated with Cytoscape (version 3.9.1), while candidate genes were ranked based on their degree centrality values calculated by the cytoHubba plugin. Hub genes were defined as those with the top 10 highest degree values.

## 3 Results

### 3.1 IBD causally affects brain cortical structure

We obtained robust instruments with average F-values surpassing 300 as genetic substitutes for IBD, UC, and CD. We then utilized PhenoScanner to detect and eliminate genetic variants shared with confounders. Ultimately, we employed 115, 69, and 98 SNPs to genetically predict IBD, UC, and CD (**Supplementary Tables S3**-**S5**). An outlier SNP, rs72924296, was excluded before estimating the causality between IBD and the globally adjusted TH of the postcentral gyrus. No outliers were detected in other phenotypic pairs. Details of SNPs used in every MR analysis are presented in **Supplementary Table S6**.

**Figure 2:**
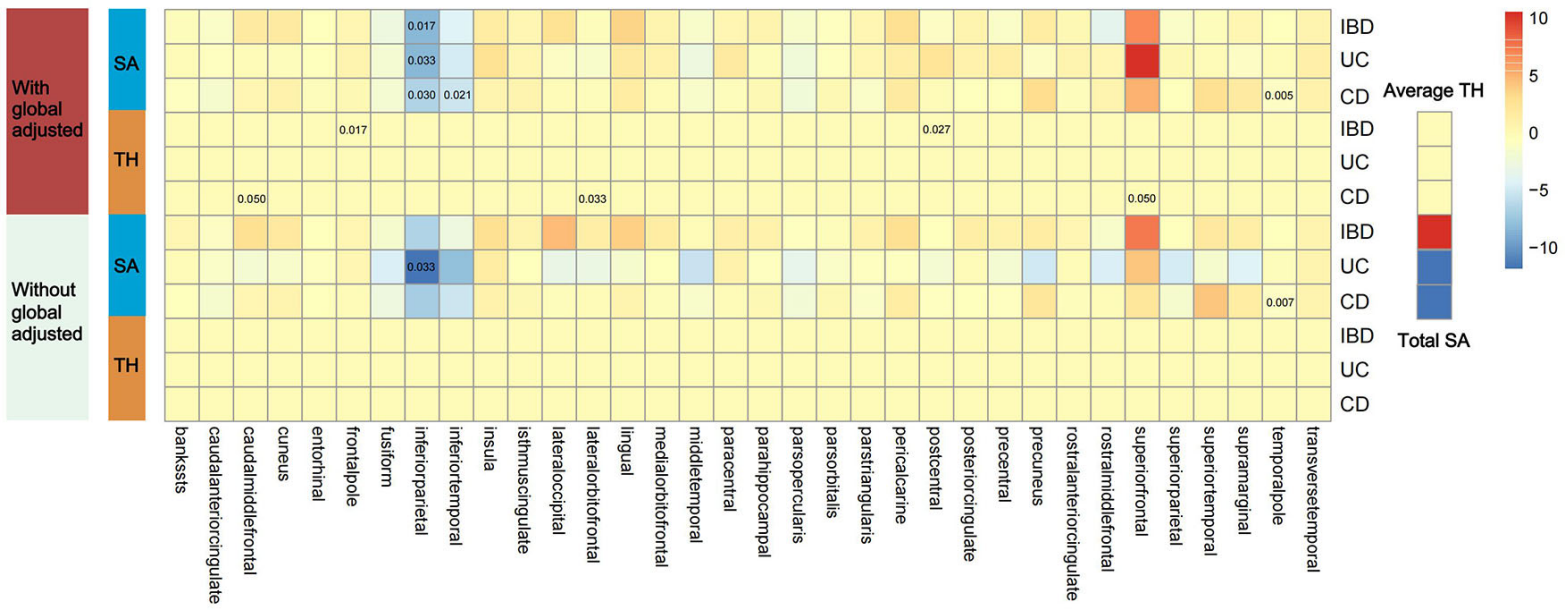
Presentation of IVW estimates derived from the IBD–cortex relationship. The color of each block represents the IVW-produced beta values, and the 12 numbers printed on the blocks are IVW-generated *P*-values. IVW, inverse-variance weighted; IBD, inflammatory bowel disease; UC, ulcerative colitis; CD, Crohn’s disease; SA, surface area; TH, thickness.

**Figure 3:**
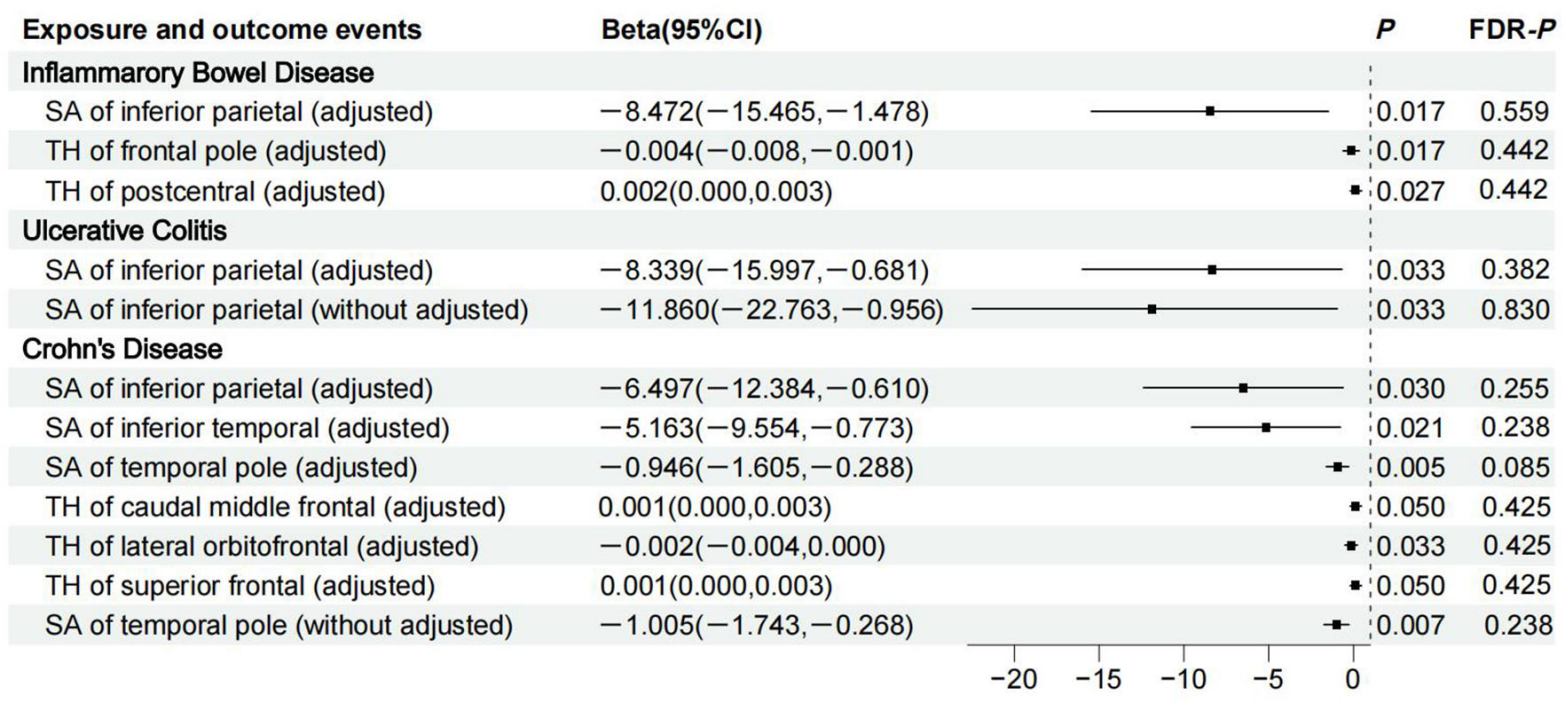
Details of causality between 12 phenotype pairs. IVW estimates from significant and suggestive causality between IBD, UC, and CD and regionally cortical SA and TH. IVW, inverse-variance weighted; IBD, inflammatory bowel disease; UC, ulcerative colitis; CD, Crohn’s disease; SA, surface area; TH, thickness; CI, confidence interval; FDR, false discovery rate.

**Figure 2** shows 12 significant and suggestive IVW-derived estimates. Following FDR correction, we realized that CD significantly decreased the globally adjusted SA of the temporal pole gyrus (β = −0.946 mm^2^, *P* = 0.005, FDR-*P* = 0.085). We have also identified 11 suggestive findings indicating regionally cortical variations induced by chronic colitis with IVW-derived *P*-values < 0.05. In terms of the affected areas, we observed that the SA was reduced in three functional gyri (inferior parietal, inferior temporal, and temporal pole) as well as a variably changed TH in the frontal pole, postcentral, caudal middle frontal, lateral orbitofrontal, and superior frontal (**Figure 3**). Our research findings showed that IBD and CD tended to promote changes in the SA and TH across distinct brain regions, whereas UC primarily affected the inferior parietal lobe by diminishing its SA. **Supplementary Figure S1** presents scatter plots illustrating the causal relationships.

Due to regional interactions, the structural variations of the entire cortex triggered by IBD, UC, and CD were found to be offset. No genetic evidence supported a causal relationship between IBD and the total SA or the average TH (β_SA_ = 27.885 mm^2^, *P*_SA_ = 0.810; β_TH_ = 1.793 × 10^−4^ mm, *P*_TH_ = 0.846). Genetic analyses suggested that neither UC nor CD had significant effects on the global SA and TH measurements (β_SA_ = −140.892 mm^2^, *P*_SA_ = 0.387 for UC; β_TH_ = 5.812 × 10^−5^ mm, *P*_TH_ = 0.953 for UC; β_SA_ = −40.268 mm^2^, *P*_SA_ = 0.715 for CD; β_TH_ = −0.001 mm, *P*_TH_ = 0.428 for CD).

All MR-Egger intercept *P*-values exceeding 0.05 and nearly deviation-free leave-one-out plots indicated that horizontal pleiotropy was effectively controlled, providing evidence for a direct causal relationship between the gut and the cerebral cortex (**Supplementary Figure S2**). We observed heterogeneity in the causal effects of IBD and CD on the postcentral and superior frontal gyri (**Supplementary Table S7**), which was deemed acceptable given the use of a random-effects model. Visually symmetric funnel plots provided evidence against directional pleiotropy (**Supplementary Figure S3**) (23). The power of MR analyses in different pairs was 100% at an alpha rate of 5%.

### 3.2 Genetic architecture mediating the causal effects of IBD

We identified dominant instrumental loci in IBD–cortex causality and mapped them to the gene database. The distribution of 1,126 instruments is shown in detail in **Supplementary Figure S4**. After removing duplicates, we finally matched 195 independent SNPs to 140 independent genes with the highest V2G scores (**Supplementary Table S8**).

**Figure 4:**
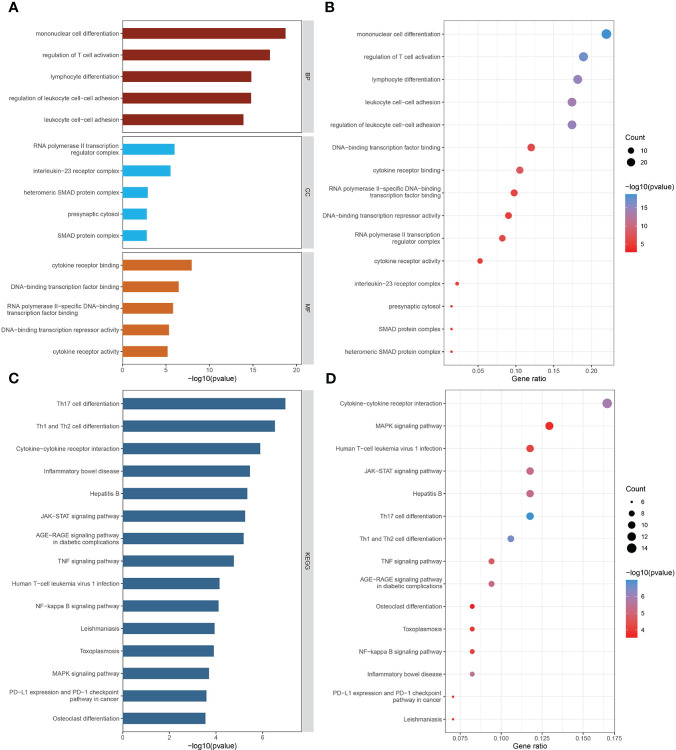
The biological functions of mediator genes. **(A, B)** The top 15 GO terms are presented in three categories: BP, CC, and MF. **(C, D)** The top 15 KEGG-enriched pathways are demonstrated. GO-term and KEGG-pathway enrichment analysis was conducted based on overrepresentation analysis, while the background gene list was the entire human genome. GO, Gene Ontology; BP, biological process; CC, cellular component; MF, molecular function; KEGG, Kyoto Encyclopedia of Genes and Genomes.

GO biological process analysis indicated that these mediators were mainly concentrated in “mononuclear cell differentiation,” “regulation of T cell activation,” “lymphocyte differentiation,” “regulation of leukocyte cell-cell adhesion,” and “leukocyte cell-cell adhesion” (**Figures 4A, B**). In GO cellular component analysis, causal genes were particularly enriched in “RNA polymerase II transcription regulator complex,” “interleukin-23 receptor complex,” “heteromeric SMAD protein complex,” “SMAD protein complex,” and “presynaptic cytosol.” Regarding GO molecular function analysis, the top 5 significantly enriched terms were “cytokine receptor binding,” “DNA-binding transcription factor binding,” “RNA polymerase II-specific DNA-binding transcription factor binding,” “DNA-binding transcription repressor activity,” and “cytokine receptor activity.” The critical KEGG-enriched pathways were “Th17 cell differentiation,” “Th1 and Th2 cell differentiation,” “Cytokine−cytokine receptor interaction,” “Inflammatory bowel disease,” and “Hepatitis B” (**Figures 4C, D**).

**Figure 5:**
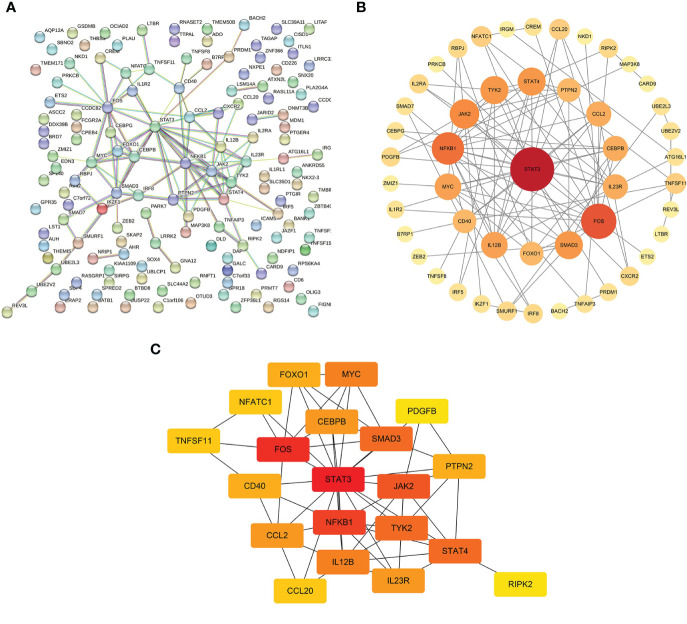
Interactions of mediator genes and hub gene identification. **(A)** Interaction network of target genes was constructed using the STRING database. **(B)** After excluding the isolated genes, other genes are ranked based on degree values and listed in descending order of importance, reflected in circle size and color shade, from red to orange to yellow. **(C)** Genes with the top 20 highest degree values are listed, of which hub genes are *STAT3*, *FOS*, *NFKB1*, *JAK2*, *STAT4*, *TYK2*, *SMAD3*, *IL12B*, *MYC*, and *CCL2*. STRING, Search Tool for the Retrieval of Interacting Genes.

The interaction networks demonstrated interconnections among the variant-associated genes (**Figure 5A**). Degree centrality values were calculated (**Figure 5B**), and the hub genes identified were *STAT3*, *FOS*, *NFKB1*, *JAK2*, *STAT4*, *TYK2*, *SMAD3*, *IL12B*, *MYC*, and *CCL2* (**Figure 5C**). The shared genetic architecture between IBD and the cerebral cortex was implicated in cytokine regulation and interaction, immune cell differentiation and activation, and immune response modulation.

## 4 Discussion

Despite the high stability and heritability of structural parameters during cortical development, multiple genetic and environmental factors likely influence the postnatal remodeling of the cerebral cortex. In our study, we utilized genetic variants obtained from large-scale GWAS datasets to disclose the promoting impact of IBD, UC, and CD on the altered cortical SA and TH. This ongoing project elucidates the regionally specific morphological changes of the human brain induced by IBD, UC, and CD and their underlying mechanisms using MR and bioinformatics approaches. This research provides a theoretical foundation for comprehending the higher prevalence of neuropsychiatric disorders and enriches our knowledge of the gut–brain axis.

MR estimates suggest that CD significantly reduces the SA of the temporal pole, a region long associated with socioemotional function. We have also identified a potentially decreased SA of the inferior parietal lobe in individuals with IBD, UC, and CD. It is reported that anomalies in the anatomical structure of the temporal pole can lead to unstable mood states (24). The inferior parietal lobe is known to play a crucial role in attention, language, and social functioning, serving as a central neural substrate for various cognitive operations (25). General cognitive ability is positively associated with an expanded SA of the evolutionary inferior parietal region (26), while a decreased cortical SA indicates a higher susceptibility to neuropsychiatric conditions (27–29). Our findings have shed light on the role of cortical reshaping in neuropsychiatric disorders induced by intestinal inflammation, encouraging the clinical utility of brain MRI. However, a previous observation has shown that a thinner TH of the inferior parietal cortex was observed in populations at greater risk of psychosis rather than a diminished SA (30). This phenomenon may be attributed to a heterogeneous population without definitive psychiatric diagnoses.

The theory of radial units posits that increased cell number resulting from neurogenic divisions is responsible for cortical TH growth (31). We found that IBD thickened the postcentral cortex, which seems complicated to understand, since the region is traditionally known as a crucial somatosensory cortex perceiving general bodily rather than visceral sensations (32). The postcentral cortex is believed to contain a visceral information region in rats (33). Furthermore, visceral perception has been confirmed to be directly relevant to regional activity and information flow around the postcentral gyrus (34). Therefore, visceral perception seems structurally or functionally relevant to the postcentral gyrus. We then hypothesize that prolonged mucosal inflammation in patients with IBD may lead to enhanced sensory input from the viscera, further activating neurogenic divisions and thickening the postcentral gyrus. Compared with patients with irritable bowel syndrome and healthy controls, the TH of the postcentral was considerably increased and positively associated with symptom duration in patients with colitis, thus supporting our proposed hypotheses (35). Meanwhile, we assume that a thickened postcentral may not be the primary cause of neuropsychiatric disorders in IBD patients as what constitutes the underlying pathophysiological mechanisms of cognitive impairment is its thinning structure (36). Further investigations are required to determine whether changes in specific cortices, such as the inferior parietal and postcentral regions, are implicated in psychosis triggered by chronic colitis.

Genetic overlap between IBD and the cerebral cortex accounts for alterations in cortical structure. A significant portion (40%) of identified hub genes were enriched in the Janus kinase-signal transducer and activator of transcription (JAK-STAT) signaling pathway, which is known to disrupt intestinal homeostasis and contribute to the development of IBD by generating multiple cytokines (37). In the brain, the aberrantly activated JAK-STAT pathway regulates pro-inflammatory gene transcription and elicits neuroinflammation (38). We propose that neuroinflammation, triggered by the JAK-STAT pathway, is a crucial agent responsible for the observed causality. Chronic gut inflammation resulting from mucosal immunity promotes the translocation of inflammatory mediators from peripheral tissues to the central nervous system, where neuroglial cells are recruited and induce neuroinflammation (39, 40). Neuroinflammation, in turn, significantly downregulates the expression of brain-derived neurotrophic factors, leading to neuronal degeneration, apoptosis, and dystrophy, ultimately resulting in altered cortical structure (41). The identification of *FOS* and *NFKB1* in the interaction network supports our assumptions. As an immediate early gene, the expression of *FOS* indicates neuronal activation to multiple stimuli (42), which has also been confirmed as a master regulator of autoimmunity and inflammation in the central nervous system (43). Given that *NFKB1* fuels neuroinflammation (44), its presence emphasizes the role of neuroinflammation in cortical morphology changes.

Other genetic loci in the gene network offer novel insights into mechanisms of cortical alterations. Genes such as *CEBPB*, *PTPN2*, and *NFATC1* exert neuroprotective effects by alleviating inflammatory responses and facilitating neurological function repair (45–47). *PDGFB* is a protein-encoding gene responsible for maintaining the integrity of the blood–brain barrier by activating pericytes, and its mutation facilitates the neuroinflammatory cascade in the brain (48). Additionally, mutant *PDGFB* has been associated with a neurodegenerative condition characterized by extensive calcification of brain areas, including the cerebral cortex (49).

This study innovatively adopted a combination of MR analysis, functional enrichment, and protein–protein interaction network to investigate the correlation between IBD and the human cortex, offering novel insights into IBD-induced brain dysfunction. Due to the differential formation pattern of UC and CD, we have implemented genetic instruments that independently predicted IBD, UC, and CD, yielding more precise estimates. The most effective genetic instruments correlated with exposure events with F-statistics over 10 were selected, and we avoided disruptions from possible confounders in every causal analysis. These considerations ensure the robustness and reliability of the MR estimates presented in the study.

However, several limitations should be acknowledged. Firstly, due to the utilization of summary statistics, stratified discussions based on age, sex, disease activity, and subjective perception score were not feasible. Secondly, target genes can only be qualitatively analyzed due to the lack of expression data, thereby impeding measuring their upregulation and downregulation within the pathways. Finally, this study focused on individuals of European heritage; therefore, our observations could be more conclusive if supplemented with large-scale studies on individuals from diverse ethnic backgrounds.

## 5 Conclusion

By utilizing GWAS summary statistics, we have shed light on the causality between intestinal inflammation and altered cortical morphology, thereby facilitating the application of brain MRI in patients with IBD. Bioinformatics analyses showed that neuroinflammation-induced neuronal damage and impaired neurological repair likely trigger cortical atrophy, whereas continuous nociceptive input increases cortical TH. Further studies on the causality between IBD and cortex as well as disease-associated mechanisms are necessary for future advancement.

## Data availability statement

All data are publicly available. The summarized inflammatory bowel disease data adopted in this paper are available from the GWAS Catalog (https://www.ebi.ac.uk/gwas/summary-statistics). The meta-analytic brain structure data presented in this paper are available from the ENIGMA consortium website (http://enigma.ini.usc.edu/research/download-enigma-gwas-results). Operation codes of two-sample MR could be obtained from the corresponding author upon a reasonable request.

## Ethics statement

Our study only extracted GWAS summary statistics from published studies, which were also publicly accessible. No extra ethical approval or informed consent was required.

## Author contributions

Research conception and design: YO, SH, and YP. Data collection and analysis: SH and YP. Figure and table production: SH, YP, and XC. Data verification: YO and XC. First drafting of article: SH and YP. All authors have revised the article and approved the submission

## References

[1] Chang JT. Pathophysiology of inflammatory bowel diseases. N Engl J Med (2020) 383(27):2652–64. doi: 10.1056/NEJMra2002697 33382932

[2] Kaplan GG Windsor JW. The four epidemiological stages in the global evolution of inflammatory bowel disease. Nat Rev Gastroenterol Hepatol (2021) 18(1):56–66. doi: 10.1038/s41575-020-00360-x 33033392 PMC7542092

[3] Barberio B Zamani M Black CJ Savarino EV Ford AC. Prevalence of symptoms of anxiety and depression in patients with inflammatory bowel disease: A systematic review and meta-analysis. Lancet Gastroenterol Hepatol (2021) 6(5):359–70. doi: 10.1016/s2468-1253(21)00014-5 33721557

[4] Kim GH Lee YC Kim TJ Kim ER Hong SN Chang DK. Risk of neurodegenerative diseases in patients with inflammatory bowel disease: A nationwide population-based cohort study. J Crohns Colitis (2022) 16(3):436–43. doi: 10.1093/ecco-jcc/jjab162 34499125

[5] Morais LH Schreiber HL IV Mazmanian SK. The gut microbiota-brain axis in behaviour and brain disorders. Nat Rev Microbiol (2021) 19(4):241–55. doi: 10.1038/s41579-020-00460-0 33093662

[6] Bisgaard TH Allin KH Keefer L Ananthakrishnan AN Jess T. Depression and anxiety in inflammatory bowel disease: epidemiology, mechanisms and treatment. Nat Rev Gastroenterol Hepatol (2022) 19(11):717–26. doi: 10.1038/s41575-022-00634-6 35732730

[7] Heydarpour P Rahimian R Fakhfouri G Khoshkish S Fakhraei N Salehi-Sadaghiani M. Behavioral despair associated with a mouse model of crohn's disease: role of nitric oxide pathway. Prog Neuropsychopharmacol Biol Psychiatry (2016) 64:131–41. doi: 10.1016/j.pnpbp.2015.08.004 26268932

[8] Zonis S Pechnick RN Ljubimov VA Mahgerefteh M Wawrowsky K Michelsen KS. Chronic intestinal inflammation alters hippocampal neurogenesis. J Neuroinflamm (2015) 12:65. doi: 10.1186/s12974-015-0281-0 PMC4403851 25889852

[9] Zhang S Chen F Wu J Liu C Yang G Piao R. Regional gray matter volume changes in brains of patients with ulcerative colitis. Inflammation Bowel Dis (2022) 28(4):599–610. doi: 10.1093/ibd/izab252 34734248

[10] Goodyear BG Heidari F Ingram RJM Cortese F Sharifi N Kaplan GG. Multimodal brain mri of deep gray matter changes associated with inflammatory bowel disease. Inflammation Bowel Dis (2022) 29(3):405–16. doi: 10.1093/ibd/izac089 PMC9977255 35590449

[11] Emdin CA Khera AV Kathiresan S. Mendelian randomization. Jama (2017) 318(19):1925–6. doi: 10.1001/jama.2017.17219 29164242

[12] Sekula P Del Greco MF Pattaro C Köttgen A. Mendelian randomization as an approach to assess causality using observational data. J Am Soc Nephrol (2016) 27(11):3253–65. doi: 10.1681/asn.2016010098 PMC5084898 27486138

[13] Gong W Guo P Li Y Liu L Yan R Liu S. Role of the gut-brain axis in the shared genetic etiology between gastrointestinal tract diseases and psychiatric disorders: A genome-wide pleiotropic analysis. JAMA Psychiatry (2023) 80(4):360–70. doi: 10.1001/jamapsychiatry.2022.4974 PMC9909581 36753304

[14] Liu JZ van Sommeren S Huang H Ng SC Alberts R Takahashi A. Association analyses identify 38 susceptibility loci for inflammatory bowel disease and highlight shared genetic risk across populations. Nat Genet (2015) 47(9):979–86. doi: 10.1038/ng.3359 PMC4881818 26192919

[15] Buniello A MacArthur JAL Cerezo M Harris LW Hayhurst J Malangone C. The nhgri-ebi gwas catalog of published genome-wide association studies, targeted arrays and summary statistics 2019. Nucleic Acids Res (2019) 47(D1):D1005–d12. doi: 10.1093/nar/gky1120 PMC6323933 30445434

[16] Grasby KL Jahanshad N Painter JN Colodro-Conde L Bralten J Hibar DP. The genetic architecture of the human cerebral cortex. Science (2020) 367(6484):eaay6690. doi: 10.1126/science.aay6690 32193296 PMC7295264

[17] Pierce BL Ahsan H Vanderweele TJ. Power and instrument strength requirements for mendelian randomization studies using multiple genetic variants. Int J Epidemiol (2011) 40(3):740–52. doi: 10.1093/ije/dyq151 PMC3147064 20813862

[18] Davies NM Holmes MV Davey Smith G. Reading mendelian randomisation studies: A guide, glossary, and checklist for clinicians. Bmj (2018) 362:k601. doi: 10.1136/bmj.k601 30002074 PMC6041728

[19] Burgess S Thompson SG. Interpreting findings from mendelian randomization using the mr-egger method. Eur J Epidemiol (2017) 32(5):377–89. doi: 10.1007/s10654-017-0255-x PMC5506233 28527048

[20] Bowden J Davey Smith G Haycock PC Burgess S. Consistent estimation in mendelian randomization with some invalid instruments using a weighted median estimator. Genet Epidemiol (2016) 40(4):304–14. doi: 10.1002/gepi.21965 PMC4849733 27061298

[21] Hartwig FP Davey Smith G Bowden J. Robust inference in summary data mendelian randomization *via* the zero modal pleiotropy assumption. Int J Epidemiol (2017) 46(6):1985–98. doi: 10.1093/ije/dyx102 PMC5837715 29040600

[22] Burgess S Butterworth A Thompson SG. Mendelian randomization analysis with multiple genetic variants using summarized data. Genet Epidemiol (2013) 37(7):658–65. doi: 10.1002/gepi.21758 PMC4377079 24114802

[23] Burgess S Davey Smith G Davies NM Dudbridge F Gill D Glymour MM. Guidelines for performing mendelian randomization investigations. Wellcome Open Res (2019) 4:186. doi: 10.12688/wellcomeopenres.15555.2 32760811 PMC7384151

[24] Olson IR Plotzker A Ezzyat Y. The enigmatic temporal pole: A review of findings on social and emotional processing. Brain (2007) 130(7):1718–31. doi: 10.1093/brain/awm052 17392317

[25] Numssen O Bzdok D Hartwigsen G. Functional Specialization within the Inferior Parietal Lobes across Cognitive Domains. Elife (2021) 10:e63591. doi: 10.7554/eLife.63591 33650486 PMC7946436

[26] Vuoksimaa E Panizzon MS Chen CH Fiecas M Eyler LT Fennema-Notestine C. Is bigger always better? The importance of cortical configuration with respect to cognitive ability. Neuroimage (2016) 129:356–66. doi: 10.1016/j.neuroimage.2016.01.049 PMC4838639 26827810

[27] Campos AI Thompson PM Veltman DJ Pozzi E van Veltzen LS Jahanshad N. Brain correlates of suicide attempt in 18,925 participants across 18 international cohorts. Biol Psychiatry (2021) 90(4):243–52. doi: 10.1016/j.biopsych.2021.03.015 PMC8324512 34172278

[28] Yang H Xu H Li Q Jin Y Jiang W Wang J. Study of brain morphology change in alzheimer's disease and amnestic mild cognitive impairment compared with normal controls. Gen Psychiatr (2019) 32(2):e100005. doi: 10.1136/gpsych-2018-100005 31179429 PMC6551438

[29] Wei X Wang Z Zhang M Li M Chen YC Lv H. Brain surface area alterations correlate with gait impairments in parkinson's disease. Front Aging Neurosci (2022) 14:806026. doi: 10.3389/fnagi.2022.806026 35153730 PMC8828503

[30] Del Re EC Stone WS Bouix S Seitz J Zeng V Guliano A. Baseline cortical thickness reductions in clinical high risk for psychosis: brain regions associated with conversion to psychosis versus non-conversion as assessed at one-year follow-up in the shanghai-at-risk-for-psychosis (Sharp) study. Schizophr Bull (2021) 47(2):562–74. doi: 10.1093/schbul/sbaa127 PMC8480195 32926141

[31] Rakic P. Specification of cerebral cortical areas. Science (1988) 241(4862):170–6. doi: 10.1126/science.3291116 3291116

[32] Kropf E Syan SK Minuzzi L Frey BN. From anatomy to function: the role of the somatosensory cortex in emotional regulation. Braz J Psychiatry (2019) 41(3):261–9. doi: 10.1590/1516-4446-2018-0183 PMC6794131 30540029

[33] Ito S. Visceral region in the rat primary somatosensory cortex identified by vagal evoked potential. J Comp Neurol (2002) 444(1):10–24. doi: 10.1002/cne.10120 11835179

[34] Nan J Yang W Meng P Huang W Zheng Q Xia Y. Changes of the postcentral cortex in irritable bowel syndrome patients. Brain Imaging Behav (2020) 14(5):1566–76. doi: 10.1007/s11682-019-00087-7 30927201

[35] Hong JY Labus JS Jiang Z Ashe-Mcnalley C Dinov I Gupta A. Regional neuroplastic brain changes in patients with chronic inflammatory and non-inflammatory visceral pain. PloS One (2014) 9(1):e84564. doi: 10.1371/journal.pone.0084564 24416245 PMC3885578

[36] Zhang W Du JL Fang XY Ni LY Zhu YY Yan W. Shared and distinct structural brain alterations and cognitive features in drug-naïve schizophrenia and bipolar disorder. Asian J Psychiatr (2023) 82:103513. doi: 10.1016/j.ajp.2023.103513 36827938

[37] Salas A Hernandez-Rocha C Duijvestein M Faubion W McGovern D Vermeire S. Jak-stat pathway targeting for the treatment of inflammatory bowel disease. Nat Rev Gastroenterol Hepatol (2020) 17(6):323–37. doi: 10.1038/s41575-020-0273-0 32203403

[38] Nabavi SM Ahmed T Nawaz M Devi KP Balan DJ Pittalà V. Targeting stats in neuroinflammation: the road less traveled! Pharmacol Res (2019) 141:73–84. doi: 10.1016/j.phrs.2018.12.004 30550953

[39] Mravec B. Pathophysiology of inflammatory bowel diseases. N Engl J Med (2021) 384(14):1377–8. doi: 10.1056/NEJMc2101562 33826832

[40] Mundt S Greter M Becher B. The cns mononuclear phagocyte system in health and disease. Neuron (2022) 110(21):3497–512. doi: 10.1016/j.neuron.2022.10.005 36327896

[41] Craig CF Filippone RT Stavely R Bornstein JC Apostolopoulos V Nurgali K. Neuroinflammation as an etiological trigger for depression comorbid with inflammatory bowel disease. J Neuroinflamm (2022) 19(1):4. doi: 10.1186/s12974-021-02354-1 PMC8729103 34983592

[42] Dempsey E Abautret-Daly Á Docherty NG Medina C Harkin A. Persistent central inflammation and region specific cellular activation accompany depression- and anxiety-like behaviours during the resolution phase of experimental colitis. Brain Behav Immun (2019) 80:616–32. doi: 10.1016/j.bbi.2019.05.007 31063848

[43] Drummond RA Swamydas M Oikonomou V Zhai B Dambuza IM Schaefer BC. Card9(+) microglia promote antifungal immunity *via* il-1β- and cxcl1-mediated neutrophil recruitment. Nat Immunol (2019) 20(5):559–70. doi: 10.1038/s41590-019-0377-2 PMC6494474 30996332

[44] Zusso M Lunardi V Franceschini D Pagetta A Lo R Stifani S. Ciprofloxacin and levofloxacin attenuate microglia inflammatory response *via* tlr4/nf-kb pathway. J Neuroinflamm (2019) 16(1):148. doi: 10.1186/s12974-019-1538-9 PMC6637517 31319868

[45] Yildirim F Foddis M Blumenau S Müller S Kajetan B Holtgrewe M. Shared and oppositely regulated transcriptomic signatures in huntington's disease and brain ischemia confirm known and unveil novel potential neuroprotective genes. Neurobiol Aging (2021) 104:122.e1–.e17. doi: 10.1016/j.neurobiolaging.2021.03.001 33875290

[46] Yoshikawa A Kamide T Hashida K Ta HM Inahata Y Takarada-Iemata M. Deletion of atf6α Impairs astroglial activation and enhances neuronal death following brain ischemia in mice. J Neurochem (2015) 132(3):342–53. doi: 10.1111/jnc.12981 25351847

[47] Wu Q Liu G Xu L Wen X Cai Y Fan W. Repair of neurological function in response to fk506 through can/nfatc1 pathway following traumatic brain injury in rats. Neurochem Res (2016) 41(10):2810–8. doi: 10.1007/s11064-016-1997-7 27386875

[48] Török O Schreiner B Schaffenrath J Tsai HC Maheshwari U Stifter SA. Pericytes regulate vascular immune homeostasis in the cns. Proc Natl Acad Sci U.S.A. (2021) 118(10):e2016587118. doi: 10.1073/pnas.2016587118 33653955 PMC7958247

[49] Cen Z Chen Y Chen S Wang H Yang D Zhang H. Biallelic loss-of-function mutations in jam2 cause primary familial brain calcification. Brain (2020) 143(2):491–502. doi: 10.1093/brain/awz392 31851307

