# Supplementary material for: Causality between inflammatory bowel disease and the cerebral cortex: insights from Mendelian randomization and integrated bioinformatics analysis
Source: Front Immunol. 2023 Jul 27;14:1175873. doi: 10.3389/fimmu.2023.1175873 (PMC10425804; doi:10.3389/fimmu.2023.1175873)

Supplementary Information

# Causality between inflammatory bowel disease and the cerebral cortex: Insights from Mendelian randomization and integrated bioinformatics analysis

Shubei He[1,2,3,4†], Ying Peng[1,2,3,4†], Xiaofang Chen[1,2,3,4], Ying Ou[5*]

**Figure S1.** Scatter plots of IBD-cerebral cortex causality.

(A) IBD and SA of inferior parietal with global adjusted. (B) IBD and TH of frontal pole with global adjusted. (C) IBD and TH of postcentral with global adjusted. (D) UC and SA of inferior parietal with global adjusted. (E) UC and SA of inferior parietal without global adjusted. (F) CD and SA of inferior parietal with global adjusted. (G) CD and SA of inferior temporal with global adjusted. (H) CD and SA of temporal pole with global adjusted. (I) CD and TH of caudal middle frontal with global adjusted. (J) CD and TH of lateral orbitofrontal with global adjusted. (K) CD and TH of superior frontal with global adjusted. (L) CD and SA of temporal pole without global adjusted.

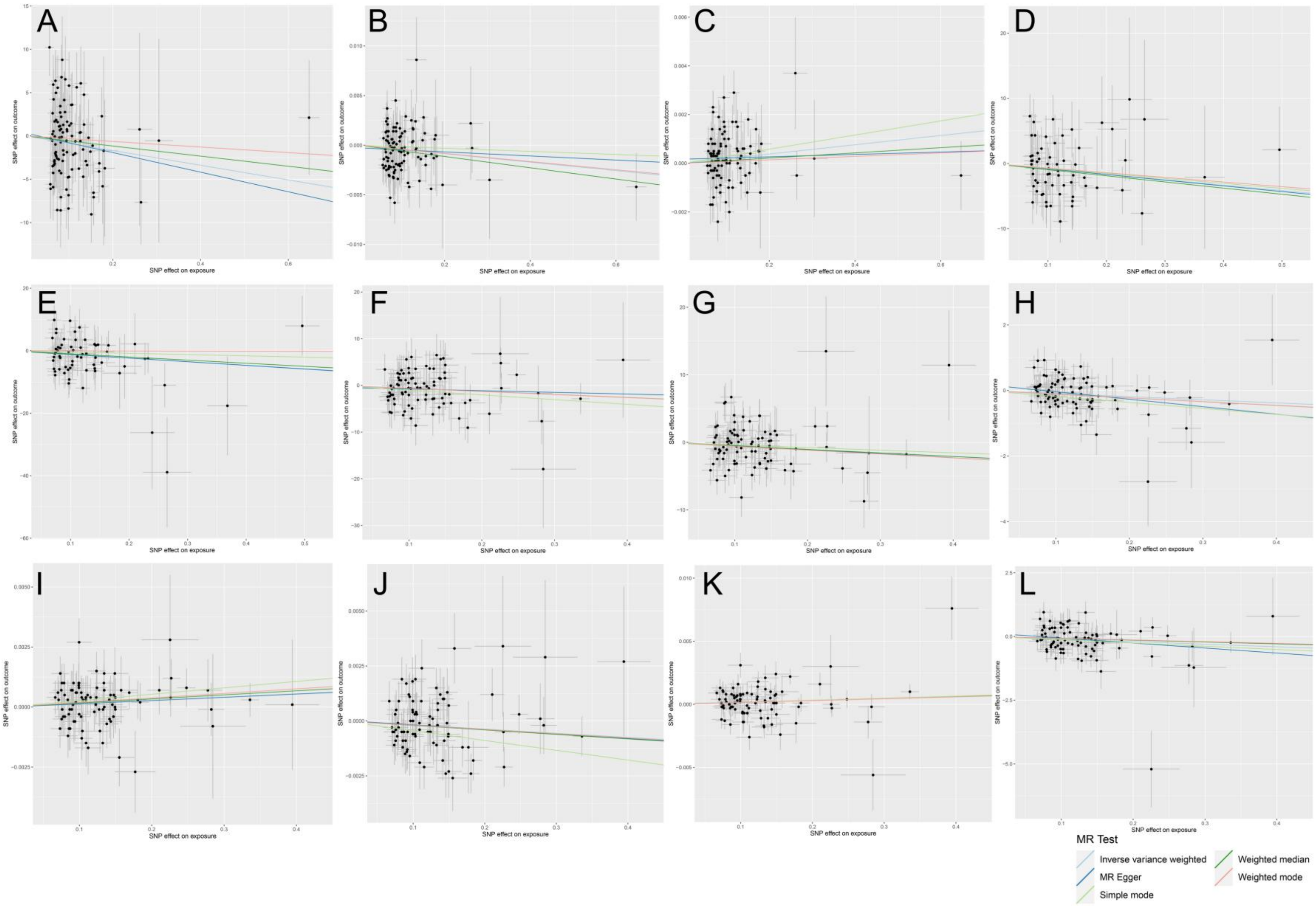

**Figure S2.** Leave-one-out plots of IBD-cerebral cortex causality.

(A) IBD and SA of inferior parietal with global adjusted. (B) IBD and TH of frontal pole with global adjusted. (C) IBD and TH of postcentral with global adjusted. (D) UC and SA of inferior parietal with global adjusted. (E) UC and SA of inferior parietal without global adjusted. (F) CD and SA of inferior parietal with global adjusted. (G) CD and SA of inferior temporal with global adjusted. (H) CD and SA of temporal pole with global adjusted. (I) CD and TH of caudal middle frontal with global adjusted. (J) CD and TH of lateral orbitofrontal with global adjusted. (K) CD and TH of superior frontal with global adjusted. (L) CD and SA of temporal pole without global adjusted.

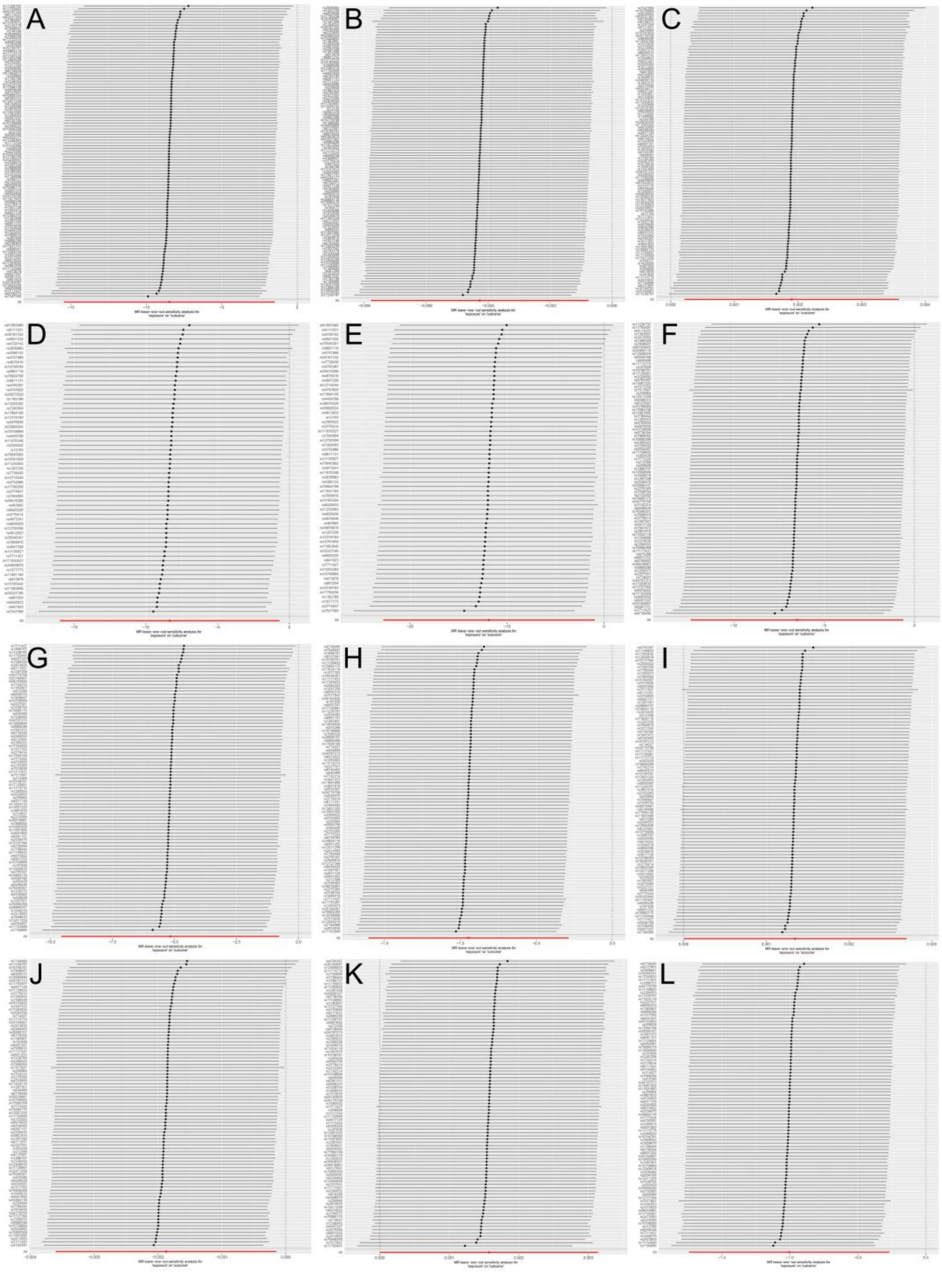

**Figure S3.** Funnel plots of IBD-cerebral cortex causality.

(A) IBD and SA of inferior parietal with global adjusted. (B) IBD and TH of frontal pole with global adjusted. (C) IBD and TH of postcentral with global adjusted. (D) UC and SA of inferior parietal with global adjusted. (E) UC and SA of inferior parietal without global adjusted. (F) CD and SA of inferior parietal with global adjusted. (G) CD and SA of inferior temporal with global adjusted. (H) CD and SA of temporal pole with global adjusted. (I) CD and TH of caudal middle frontal with global adjusted. (J) CD and TH of lateral orbitofrontal with global adjusted. (K) CD and TH of superior frontal with global adjusted. (L) CD and SA of temporal pole without global adjusted.

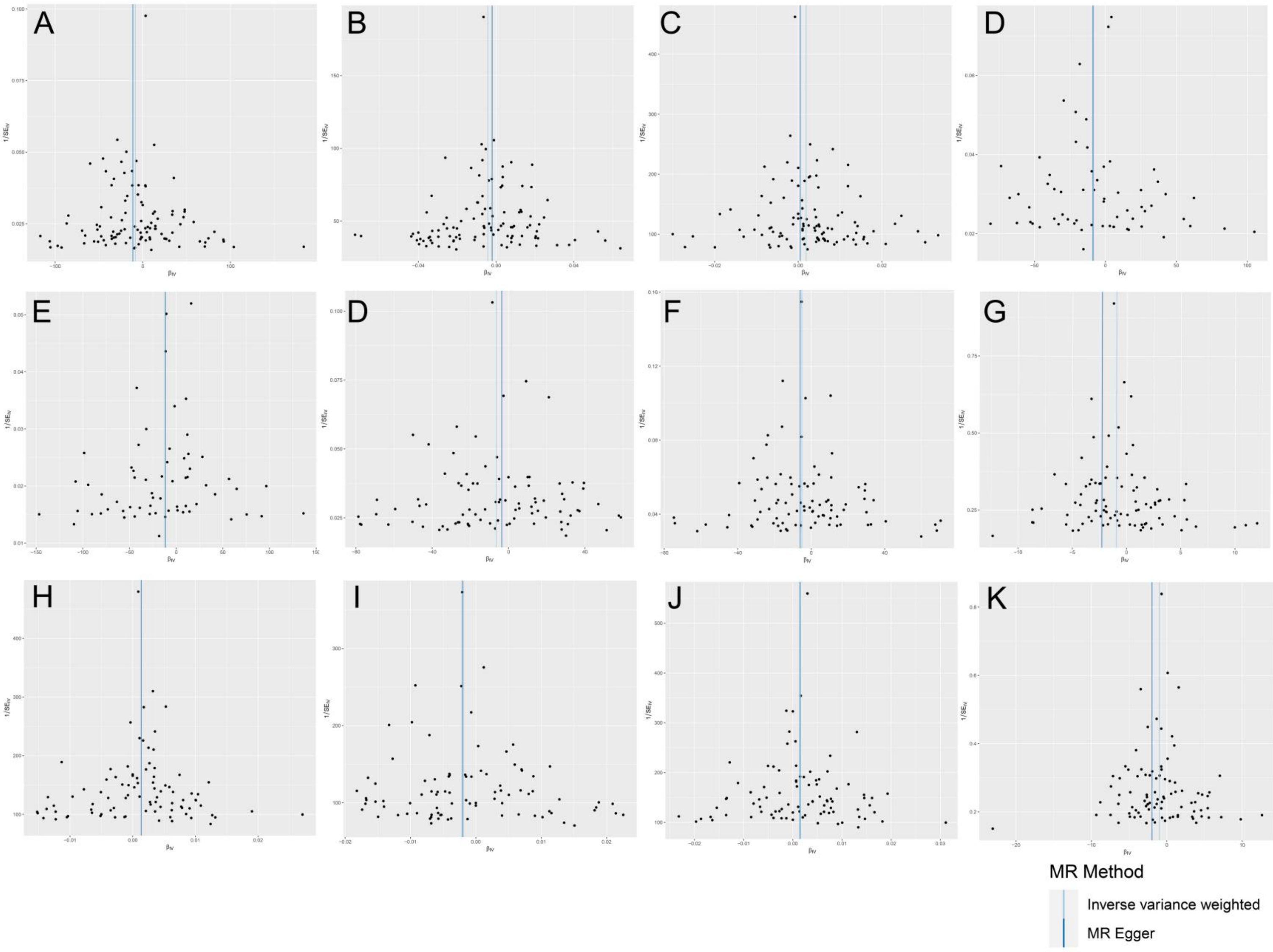

**Figure S4.** Distribution of genetic variants in 12 phenotype pairs.

The number of SNPs for each MR estimate is presented by the bars in the left corner, while the intersecting SNPs for particular events are demonstrated by the bars in the upper right and the circles below.

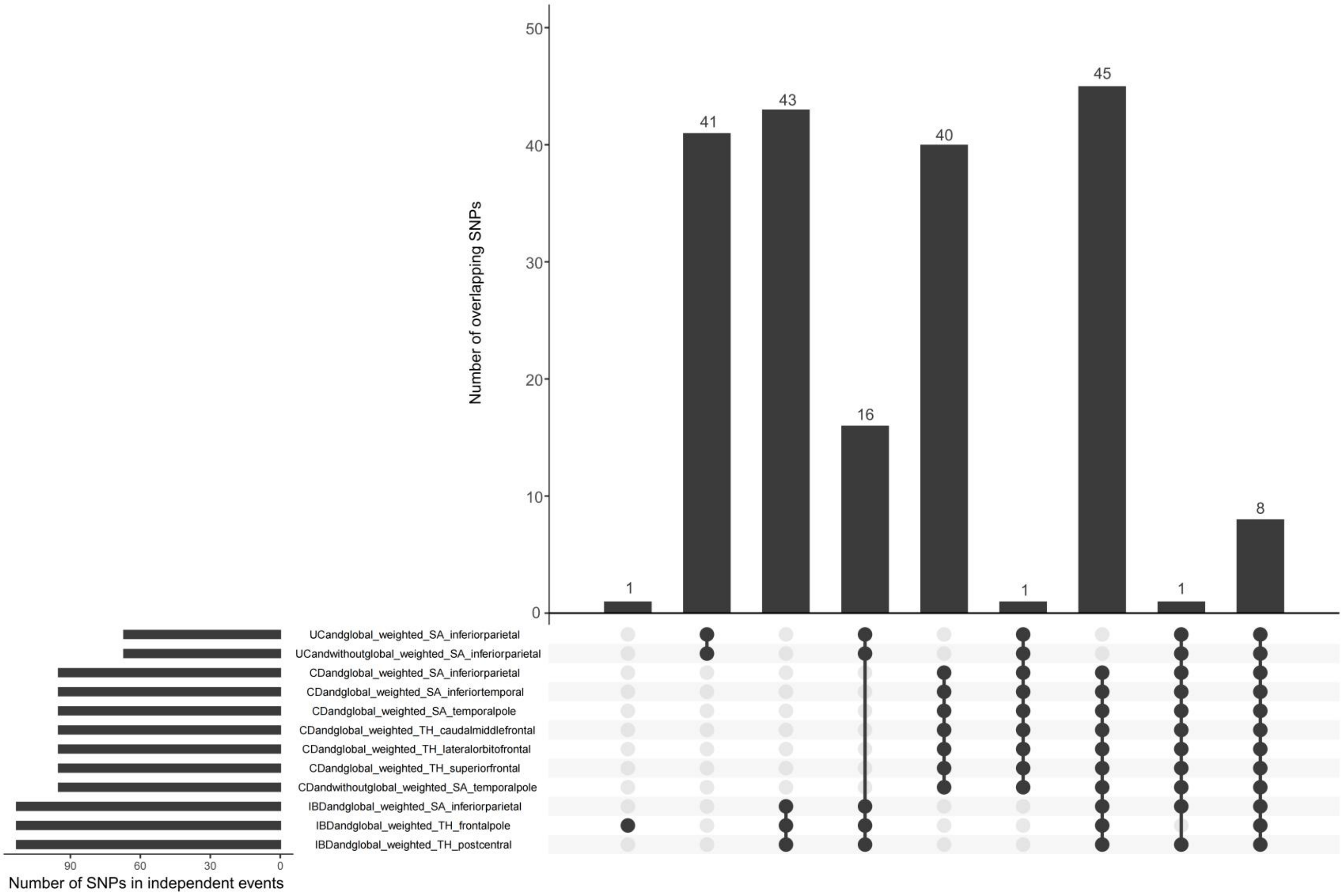

Supplement: Supplementary file 1 [file DataSheet_1.pdf]
